# Distinct gene loci control the host response to influenza H1N1 virus infection in a time-dependent manner

**DOI:** 10.1186/1471-2164-13-411

**Published:** 2012-08-20

**Authors:** Tatiana Nedelko, Heike Kollmus, Frank Klawonn, Sabine Spijker, Lu Lu, Manuela Heßman, Rudi Alberts, Robert W Williams, Klaus Schughart

**Affiliations:** 1 Department of Infection Genetics, Helmholtz Centre for Infection Research and University of Veterinary Medicine Hannover, 38124, Braunschweig, Germany; 2 Department of Bioinformatics and Statistics, Helmholtz Centre for Infection Research, Braunschweig, Germany; 3 Department of Computer Science, Ostfalia University of Applied Sciences, Wolfenbüttel, Germany; 4 Department of Anatomy and Neurobiology, University of Tennessee Health Science Center, Memphis, Tennessee, United States of America; 5 Department of Molecular and Cellular Neurobiology, Neuroscience Campus Amsterdam, Amsterdam, VU, the Netherlands; 6 Jiangsu Key Laboratory of Neuroregeneration, Nantong University, Nantong, China; 7 Nycomed GmbH, Institute for Pharmacology and Preclinical Drug Safety, Barsbuettel-Willinghusen, Germany

## Abstract

**Background:**

There is strong but mostly circumstantial evidence that genetic factors modulate the severity of influenza infection in humans. Using genetically diverse but fully inbred strains of mice it has been shown that host sequence variants have a strong influence on the severity of influenza A disease progression. In particular, C57BL/6J, the most widely used mouse strain in biomedical research, is comparatively resistant. In contrast, DBA/2J is highly susceptible.

**Results:**

To map regions of the genome responsible for differences in influenza susceptibility, we infected a family of 53 BXD-type lines derived from a cross between C57BL/6J and DBA/2J strains with influenza A virus (PR8, H1N1). We monitored body weight, survival, and mean time to death for 13 days after infection. *Qivr5* (quantitative trait for influenza virus resistance on chromosome 5) was the largest and most significant QTL for weight loss. The effect of *Qivr5* was detectable on day 2 post infection, but was most pronounced on days 5 and 6. Survival rate mapped to *Qivr5*, but additionally revealed a second significant locus on chromosome 19 (*Qivr19*). Analysis of mean time to death affirmed both *Qivr5* and *Qivr19*. In addition, we observed several regions of the genome with suggestive linkage. There are potentially complex combinatorial interactions of the parental alleles among loci. Analysis of multiple gene expression data sets and sequence variants in these strains highlights about 30 strong candidate genes across all loci that may control influenza A susceptibility and resistance.

**Conclusions:**

We have mapped influenza susceptibility loci to chromosomes 2, 5, 16, 17, and 19. Body weight and survival loci have a time-dependent profile that presumably reflects the temporal dynamic of the response to infection. We highlight candidate genes in the respective intervals and review their possible biological function during infection.

## Background

Influenza A virus represents a major health threat to humans. The 1918 H1N1 pandemic caused at about 30 to 50 million deaths [1]. Seasonal influenza epidemics cause high economic loss, morbidity and deaths every year [2]. The course and outcome of an influenza A virus infection is influenced by viral and host factors. Host risk factors, like obesity or pregnancy, became evident during the recent swine flu pandemics [3,4]. Furthermore, genetic factors in humans associated with a higher susceptibility to influenza infections and severe disease outcome have been suspected for the 1918 pandemics, as well as the H5N1 human infections [5-7]. Recently, the importance of *IFITM3* as a crucial factor for host susceptibility has been demonstrated in mice and humans [8].

The importance of host factors to host susceptibility and resistance has been demonstrated clearly in animal models. We and others have shown in mouse infection models that the susceptibility of the host to influenza A infection strongly depends on the genetic background [9-17]. In particular, DBA/2J mice are highly susceptible to many influenza A virus subtypes, including those that were directly derived from human isolates without prior adaptation to the mouse [9,13,16-18]. In contrast, C57BL/6J mice are more resistant. After infection with mouse-adapted H1N1 (PR8M virus), DBA/2J mice loss weight very rapidly and die within 5–7 days post infection (p.i.), whereas C57BL/6J mice loss weight until days 6–8 after infection and regain their initial weight by 14 days p.i. [9,18]. Viral load in the lungs of DBA/2J infected mice is much higher and lung pathology is very severe compared to infected C57BL/6J mice. Also, the production of chemokines and cytokines is much higher in DBA/2J mice [9,18].

However, the genomic regions that are responsible for the differential response after infection with H1N1 have not been determined. Therefore, we used a large family of BXD type recombinant inbred strains generated by crossing C57BL/6J (resistant) to DBA/2J (susceptible) to map genetic loci that modulate disease severity. The BXD genetic reference population (GRP) is made up of a set of progeny strains, each with a defined and fixed genetic architecture. It is one of the largest families of strains, consisting of about 80 fully inbred strains [19,20] available from the Jackson Laboratory and a new set of 80 additional lines that are still in production at the University of Tennessee. Individuals within each single strain are essentially isogenic (except for the sex chromosomes) and genotypes for the entire family, including most of the new strains, are known [21]. Genetic variation among this family has be exploited extensively in the past to systematically study the genetics of many traits (for examples of phenotypes see the GeneNetwork database [22]).

Here, we infected over 50 of the BXD strains with influenza A H1N1 virus and monitored body weight, survival, and mean time to death for the following 13 days post infection. We identified two significant and several suggestive loci peaks for all three traits. All showed a time-dependent appearance. Data mining of the intervals revealed several candidate genes, several of which may be important for the host response to influenza A virus infection.

## Results

### Susceptibility to influenza A virus after experimental infection of BXD mouse strains is highly variable

**Figure 1:**
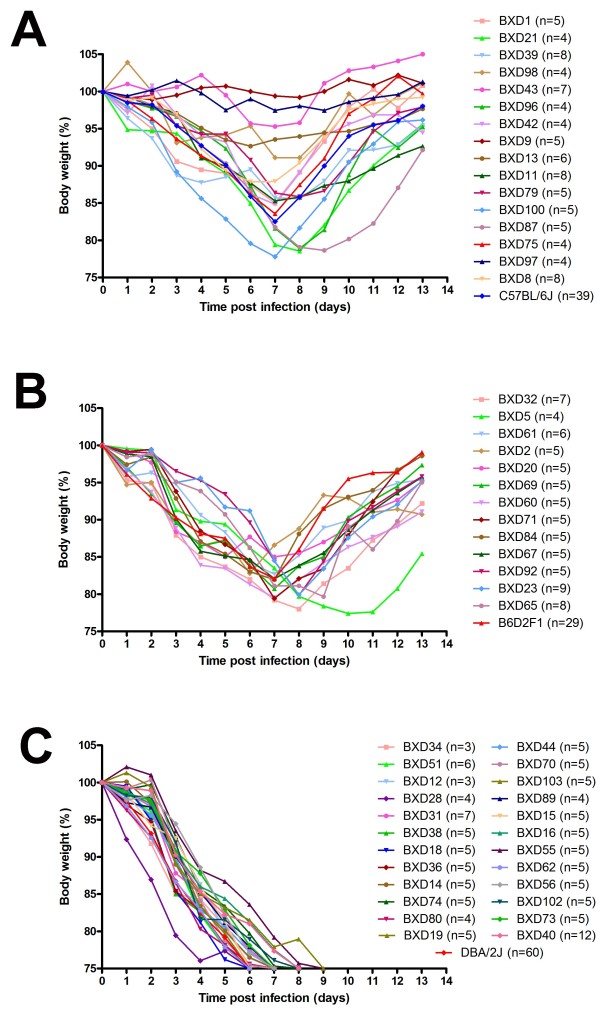
**BXD strains exhibit variable kinetics of weight loss and survival after infection with Influenza A virus.** Mice from 53 BXD and parental strains were infected intra-nasally with 2 × 10^3^ FFU of PR8 virus. Weight loss and survival of infected mice was followed over a period of 13 days. Mortality includes mice that were sacrificed because they had lost more than 25% of body weight. Three phenotypic groups can be distinguished: in the first group (**A**), all infected mice within a given BXD strain survived, in the second group (**B**) less than 50% of infected mice within a given BXD strain died, in the third group (**C**), more than 50% of infected mice within a given BXD strain died. From the weight loss curve of the second and third group it also becomes obvious that non-surviving mice were all approaching the 75% body weight loss endpoint before dying.

We infected 53 recombinant inbred strains of the BXD population plus the parental strains C57BL/6J, DBA/2J and B6D2F1 mice with mouse-adapted H1N1 virus (PR8M, H1N1 [18]) and followed body weight and survival over the next 13 days. Mice that lost more than 25% of their starting weight were sacrificed and also recorded as dead. After infection, body weight changes were highly variable between the BXD strains over the period of 13 days post infection (Figure 1, Additional file 1: Table S1). Similarly, survival rates (Additional file 2: Figure S1A, B) and mean time to death (MTTD; Additional file 2: Figure S1C) were highly variable between the different BXD strains. Furthermore, the trait percent survival showed a strain-dependent progression over time.

For body weight loss and survival, three different phenotypic response groups can be defined (Figure 1). In the first (Figure 1A), all infected mice within a strain survived, in the second (Figure 1B) a majority but not all individuals within a strain survived, and in the third group (Figure 1C), a majority died. Most remarkably, in the first group four strains—BXD9, BXD13, BXD43, BXD97—were highly resistant indicating that the infection may not cause any major pathology (Figure 1A). In contrast, BXD28 belonging to the third group, lost body weight much more rapidly than even the highly susceptible DBA/2J parent (Figure 1C). These results illustrate a large variation of responses within the BXD family.

By day 7 p.i., all infected mice had succumbed to infection in 11 strains, whereas 14 others exhibited more limited mortality (Additional file 2: Figure S1A). The incidence of mortality increased in some strains from day 8 p.i. through day 11 but not thereafter (Additional file 2: Figure S1B). For the MTTD phenotype, 17 strains showed no mortality after infection, similar to the resistant C57BL/6J parent. Three strains—BXD28, BXD18, BXD103—exhibited a MTTD that was even shorter than for the susceptible DBA/2J parent (Additional file 2: Figure S1C). Although this study was conducted over a period of approximately three years, and although mice were received from different sources and different experimenters performed the infection experiments, we did not note any significant influence of these potential confounds and cofactors.

**Figure 2:**
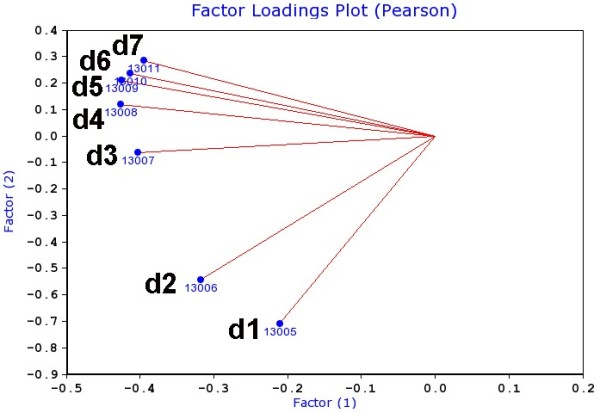
**Principal component analysis of all body weight traits reveals two distinct groups.** PCA loading plot of principal components PC1 and PC2 (Supplement Figure 2) for body weight traits form day 1 to day 7 p.i. shows two distinct groups. The variation of body weight loss at day 3 to day 7 (ID 13007 to ID 13011) are mainly represented by factor 1. On the other hand, variation of body weight loss at day 1 (ID 13005) and 2 (ID 13006) are most likely explained by factor 2.

Principal Component Analysis (PCA) of strain mean body weight loss from day 1 until day 7 was carried out to reduce the number and redundancy of related measurements, as well as to evaluate whether this set of measurements can be broken down into statistically and genetically independent processes. This analysis revealed one major component, PC1 that explains most of the weight loss among strains (more than 80%, see Additional file 3: Figure S2). This component corresponds to differences in loss from day 3 to 7 p.i. (Figure 2). In contrast, PC2 accounts for only about 10% of the variance in weight loss (Additional file 3: Figure S2). This component corresponds only to very early weight loss after virus inoculation, on days 1 and 2 p.i.(Figure 2), well before any appreciable virus replication has occurred. Thus, PC2 is highly likely to be a technical, but still interesting effect associated with the stressful procedure of intranasal inoculation and full anesthesia. PC1 most likely reflects the biologically relevant variation among strains in response to viral replication.

### Analysis of body weight loss revealed a significant QTL on chromosome 5 and several suggestive QTLs with time-dependent effects

**Figure 3:**
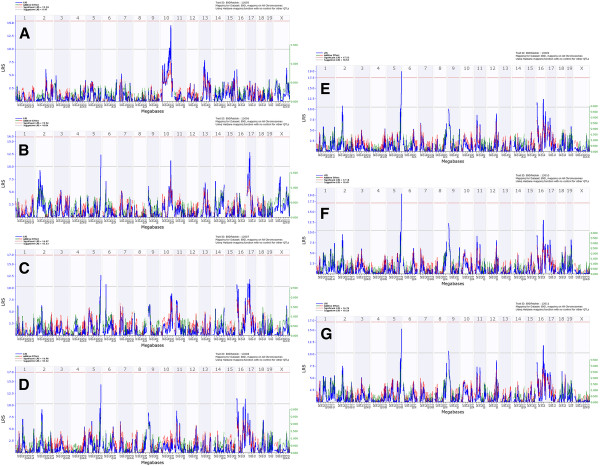
**Genome-wide linkage analysis for body weight loss after H1N1 infection identifies time-dependent QTLs.** Analysis of body weight from day 1 (**A**), day 2 (**B**), day 3 (**C**), day 4 (**D**), day 5 (**E**), day 6 (**F**) and day 7 (**G**) after PR8 infection revealed time-dependent QTLs. A significant QTL is located on chromosome 5 and suggestive QTLs are observed on chromosomes 2, 6, 9, 10, 16 and 17. Dead or euthanized mice were included for the analysis by manually setting their body weight to 75%. Interval mapping is shown across the entire genome as blue line representing the LRS scores; green line: DBA/2J alleles increase trait values; red line: C57BL/6J alleles increase trait values. Upper x-axis shows mouse chromosomes, lower x-axis shows physical map in mega bases for each chromosome, y-axis represents LRS score. The horizontal lines mark the genome-wide significant thresholds at p<0.05 (red line) and suggestive thresholds at p<0.37 (gray line).

We mapped body weight loss following infection day by day. For purposes of analysis, body weights of mice that had died or that were euthanized were assigned a weight equal to 75% of their initial weight. It should be noted that mice which died continuously lost weight and were close to 75% body weight loss before they were found dead (Figure 1B, C). After day 7, surviving mice started to gain weight (Figure 1A, B). For this reason, we limited our analyses of body weight traits to the period of day 1 to day 7 p.i. in order to avoid mixing data from dead with recovering mice. Interval mapping of body weight loss detected a significant time-dependent locus on chromosome 5 that we named ‘*QTL for influenza virus resistance on chromosome 5*’ (*Qivr5)* adopting the nomenclature proposed by [12]. This QTL exhibited genome-wide significance on days 5 (LRS: 19.0, effect size: 30%) and 6 (LRS: 19.4, effect size: 31%, Figure 3). However, the effect of *Qivr5*, although weaker, can also be detected on days 2, 3, and 4 and 7 (Figure 3). Most interestingly; the resistance allele at *Qivr5* is inherited from the nominally sensitive DBA/2J parental strain, illustrating the genetic complexity of the influenza response. Suggestive loci (LRS values between 10 and 15) map to chromosomes 2, 6, 9, proximal and distal 16, and chromosome 17. The effect of these loci was also time-dependent (Figure 3A-G).

Another suggestive QTL peak was found on chromosome 10 on day 1 (LRS: 14.1, effect size: 23%, Figure 3A). Its effect is lower at day 2 and not apparent at later days. These observations are in accordance with the PCA (Figure 2) that reveals two separate time-dependent influences on body weight variance among strains. The QTL appeared at an early time point after infection, when virus replication has just begun and strong inflammatory host responses are not yet evident [9,18,23]. These observations indicate that the effect is most likely related to the experimental protocol, namely the stress to anesthesia and intra-nasal application as well as treatment recovery. Treatment-dependent QTLs were described previously [24]. It is worth to note that mock-infection of the parental DBA/2J and C57BL/6J mice did not lead to a lasting body weight loss over a longer time interval except for a slight drop in body weight on day 1 p.i. (Additional file 4: Figure S3).

**Figure 4:**
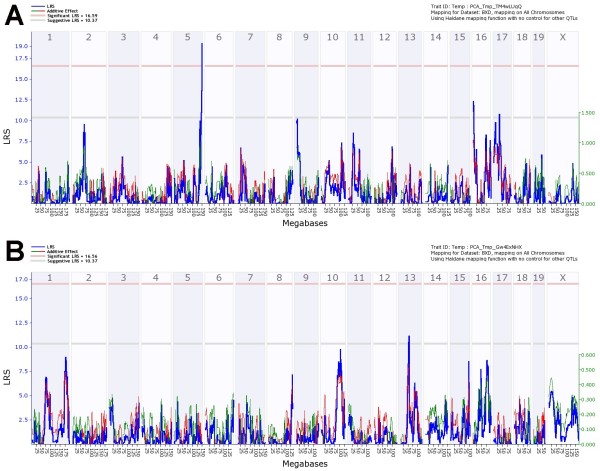
**Genome-wide linkage scan for PC1 and PC2 body weight loss.** Interval mapping of principle component PC1 (**A**) for body weight loss traits at day 1 to 7 after PR8 infection shows significant QTLs on chromosome 5 and suggestive QTLs on chromosomes 16 and 17. Interval mapping of PC2 (**B**) for body weight loss traits reveals no significant QTLs and a suggestive QTL on chromosome 13. Interval mapping is shown across the entire genome as blue line representing the LRS scores; green line: DBA/2J alleles increase trait values; red line: C57BL/6J alleles increase trait values. Upper x-axis shows mouse chromosomes, lower x-axis shows physical map in mega bases for each chromosome, y-axis represents LRS score. The horizontal lines mark the genome-wide significant thresholds at p<0.05 (red line) and suggestive thresholds at p<0.37 (gray line).

We also performed a QTL analysis for the PC1 and PC2 described above. PC1 detects a significant QTL on chromosome 5 (LRS: 19, effect size: 30%, Figure 4A) as well as suggestive QTL peaks on proximal chromosome 16 and on chromosome 17 (Figure 4A). Thus, the PC1 confirmed the significant QTL on chromosome 5 found with the daily body weight loss traits. Interval mapping of PC2 detected no significant QTL and one suggestive QTL on chromosome 13 (Figure 4B) which was not seen in any other trait.

### Survival rate and mean time to death traits confirmed the QTL on chromosome 5 and detected another significant QTL on chromosome 19

**Figure 5:**
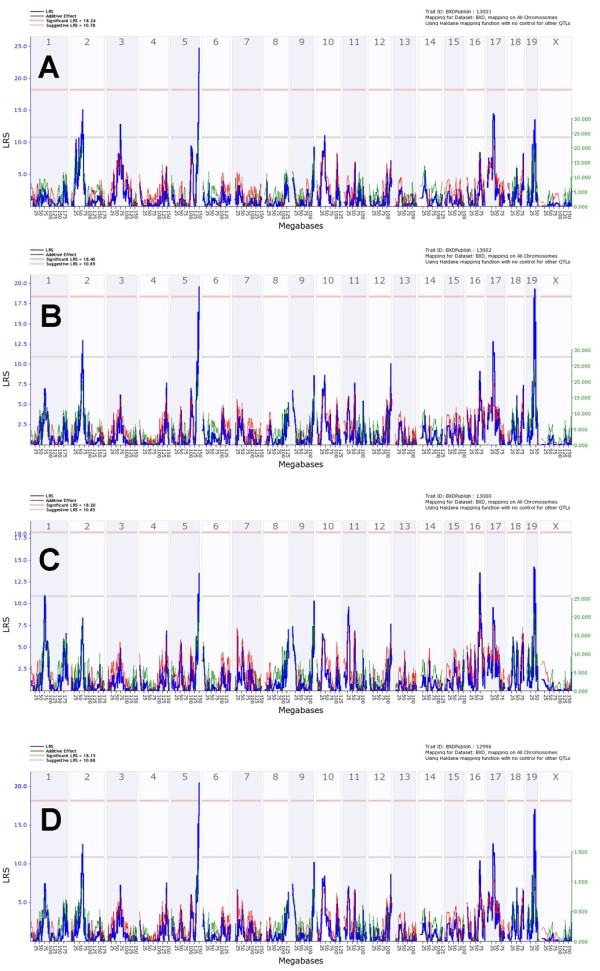
**Genome-wide linkage scan for survival rate and mean time to death confirms QTLs on chromosomes 5 and 19.** Interval mapping of survival rates (in %) after PR8 infection at day 7 (**A**), day 8 (**B**) and day 11 (**C**) show significant QTLs on chromosomes 5 and 19. Suggestive QTLs are observed on chromosomes 2, 3, 10, 16 and 17. Interval mapping using mean time to death confirmed the significant QTL on chromosome 5 and revealed suggestive QTLs on chromosomes 2, 17 and 19 (**D**). Interval mapping is shown across the entire genome as blue line representing the LRS scores; green line: DBA/2J alleles increase trait values; red line: C57BL/6J alleles increase trait values. Upper x-axis shows mouse chromosomes, lower x-axis shows physical map in mega bases for each chromosome, y-axis represents LRS score. The horizontal lines mark the genome-wide significant thresholds at p<0.05 (red line) and suggestive thresholds at p<0.37 (gray line).

The analysis of survival rate traits on days 7, 8 and 11 p.i. revealed significant peaks on chromosomes 5 and 19 and suggestive peaks on chromosomes 1, 2, 3, 10, 16 and 17 (Figure 5A-C). The effects of these QTLs were again time-dependent. *Qivr5* was significant at day 7 (LRS: 24.8, effect size: 37%) and day 8 p.i. (LRS: 19.7, effect size: 31% Figure 5A, B) and its effect was still evident from day 9 until 11 p.i. (Additional file 5: Figure S4 and Figure 5C). Similarly, another significant QTL with time-dependent effects was observed on chromosome 19 (*Qivr19*) at day 8 (LRS: 19.4; effect size: 29%) that could also be detected at day 7 and days 9–11 (Figure 5A-C and Additional file 5: Figure S4). *Qivr5* and *Qivr19* resulted from a positive influence of DBA/2J on survival, whereas *Qivr16* (distal locus) and *Qivr17-2* resulted from a positive influence of C57BL/6J alleles to increase survival. Furthermore, MTTD analysis confirmed *Qivr5* as a significant QTL (LRS: 20.5, effect size: 32%), and *Qivr19* was almost significant for this trait (Figure 5D). In addition, suggestive QTLs were found on chromosomes 2 and 17 for the MTTD trait.

In conclusion, survival and MTTD traits confirmed the significant QTL on chromosome 5, revealed an additional significant QTL on chromosome 19 and several suggestive QTLs. All QTLs showed a time-dependent effect.

### Composite interval and pair-scan mapping indicates various interactions of QTLs

**Figure 6:**
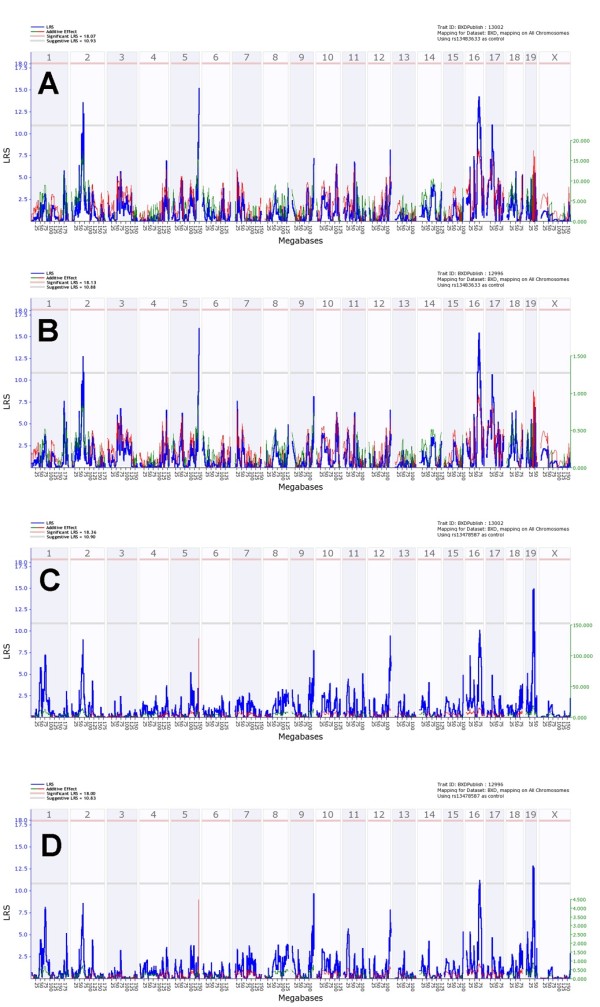
**Composite interval mapping indicates interactions of QTLs on chromosomes 5 and 19.** The influence of the markers rs13483633 on chromosome 19 (**A**, **B**) and rs13478587 on chromosome 5 (**C**, **D**) was factored out and the residual LRS were calculated. Two different traits, survival day 8 (A, C) and mean time of death (B, D) were tested by using the web tool of GeneNetwork. Interval mapping is shown across the entire genome as blue line representing the LRS scores; green line: DBA/2J alleles increase trait values; red line: C57BL/6J alleles increase trait values. Upper x-axis shows mouse chromosomes, lower x-axis shows physical map in mega bases for each chromosome, y-axis represents LRS score. The horizontal lines mark the genome-wide significant thresholds at p<0.05 (red line) and suggestive thresholds at p<0.37 (gray line).

The strongest QTLs map to chromosomes 5 and 19. We therefore performed composite interval mapping in which the contributions of these strong QTLs were factored out to reveal possible secondary QTLs. When we controlled for *Qivr19*, the linkage to distal chromosome 16 increased and became nearly significant for both survival at day 8 and MTTD (Figure 6A, B). The linkage to chromosome 2 increased slightly. However, control for the contribution of *Qivr5* did not reveal any additional loci linked to survival or MTTD traits (Figure 6C, D).

**Figure 7:**
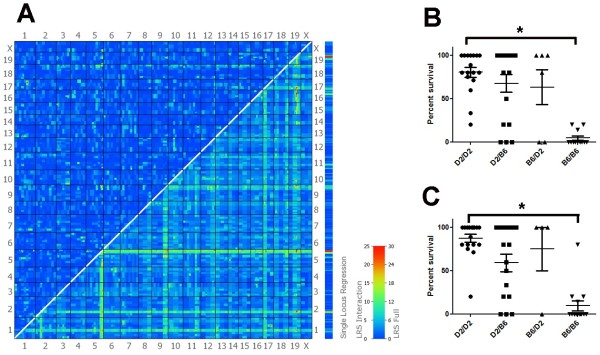
**Interactions are observed between several QTLs.** The graph displays pair-scan results for the trait percent survival at day 8 p.i. (ID13002) (**A**). The upper left half of the plot highlights any epistatic interactions, the lower right half provides a summary of LRS of the full model, representing cumulative effects of linear and non-linear terms based on the model ‘Trait Variance = Q1 + Q2 + Q1 x Q2 ’. Subsequently, BXD lines were grouped according to their allele combinations and trait values were compared between groups (**B**, **C**). The largest differences are indicated by asterisks. They are highly significant between the respective groups (p < 0.0001). For the various loci, trait values for the following markers were used: *Qivr5*: rs13478587, chromosome 9: rs6191976, *Qivr19*: rs13483633. Number of lines and p-values for *Qivr5* – chromosome 9: D2/D2: n = 17, D2/B6: n = 17, B6/D2: n = 6, B6/B6 n = 12; D2/D2 vs B6/B6: p < 0.0001, D2/B6 vs B6/B6: p = 0.003. Number of lines and p-values for *Qivr5* – *Qivr19*: D2/D2: n = 18, D2/B6: n = 16, B6/D2: n = 4, B6/B6: n = 14; p-values: D2/D2 vs B6/B6: p < 0.0001, D2/B6 vs B6/B6: p = 0.0008. Abbreviations: D2: DBA/2J, B6: C57BL/6J.

Finally, we analyzed the joint additive effects of QTLs described above as well as possible epistatic interactions among these QTLs. When using a full two-locus model (Trait Variance = Q1 + Q2 + Q1 x Q2), we found two potential interactions between *Qivr5* and a new locus on chromosome 9 as well as between Qivr5 and *Qivr19*, respectively, for the survival trait at day 8 (Figure 7A). The inclusion of an interaction term increases the LRS by 23 (Additional file 6: Figure S5). For both *Qivr5* - chromosome 9 interaction (Figure 7B) and *Qivr5* – *Qivr19* interaction (Figure 7C), the allele combination DBA/2J / DBA/2J showed highest and the combination C57BL/6J / C57BL/6J lowest survival scores. All differences were highly significant.

### Analysis of QTL regions identified several candidate genes that may contribute to host susceptibility or resistance

In total, the mapping studies revealed five QTLs on chromosomes 2, 5, distal 16, 17 and 19 (*Qivr2-2*, *Qivr5*, *Qivr16*, *Qivr17-2* and *Qivr19*) that did merit further analysis because they were consistently observed in at least two traits and exerted an effect on at least two different days p.i.

**Figure 8:**
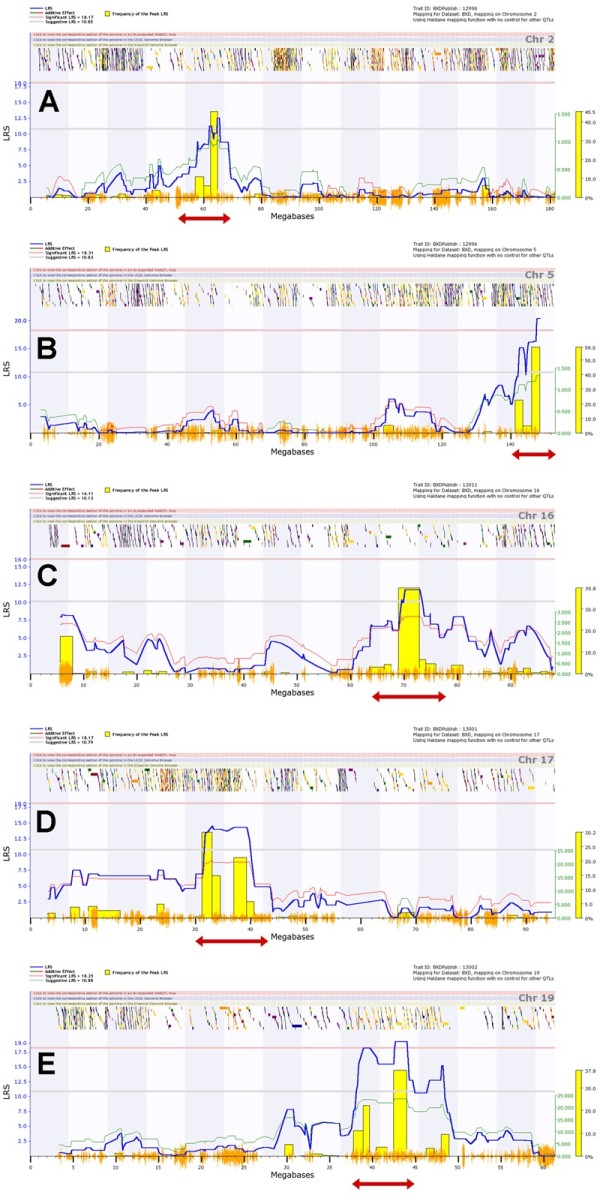
**Detailed maps of QTLs contributing to host susceptibility and resistance to PR8 influenza A infection.** Interval mapping was performed for chromosomes 2 (**A**), 5 (**B**), 16 (**C**), 17 (**D**) and 19 (**E**). The critical intervals were selected based on the peak shapes and the bootstrap signals: *Qivr2-1* (56–68 Mb), *Qivr5* (140–153 Mb), *Qivr16* (64–78 Mb), *Qivr17-2* (30–44 Mb) and *Qivr19* (37–45 Mb). The *Qivr* intervals are delineated by red arrows. The yellow bars represent the frequencies of peak LRS using bootstrap analysis. The multicolored chatters along the top of the graph are hyperlinks to sites with additional genetic and sequence information. The orange chatter along the x-axis indicates the density of SNPs present in the BXD strains. Interval mapping is shown across the entire genome as blue line representing the LRS scores; green line: DBA/2J alleles increase trait values; red line: C57BL/6J alleles increase trait values. Upper x-axis shows mouse chromosomes, lower x-axis shows physical map in mega bases for each chromosome, y-axis represents LRS score. The horizontal lines mark the genome-wide significant thresholds at p<0.05 (red line) and suggestive thresholds at p<0.37 (gray line).

For the candidate gene searches, we defined the critical regions of the QTLs manually by considering peak height, its shoulder and bootstraps as guidance (Figure 8). We then investigated these *Qivr* intervals for potential candidate genes that may be causal for the studied traits (Additional file 7: Figure S6). We first used the QTLminer tool in GeneNetwork [25] to identify all genes in the QTL intervals and to obtain associated GO terms. Next, we select all annotated genes within the QTL intervals that were expressed between day 1 and day 60 after infection of C57BL/6J mice with PR8M virus (the latter data set is derived from a separate study (Pommerenke et al., PLoS ONE, in press). Subsequently, these genes were further characterized for the following attributes: genes that were up- or up-regulated in infected lungs by at least 1.5-fold in C57BL/6J after PR8M infection, genes carrying an insertion or deletion or a non-synonymous nucleotide change in the open reading frame, genes exhibiting a cis-expression QTL in the lung [26], and genes that were differentially expressed by at least 1.5-fold in C57BL/6J and DBA/2J mice in infected lungs between day 1 and day 8 p.i. [23] and Pommerenke et al., PLoS ONE, in press). The strategy of the QTL mining is shown as flow chart in the Additional file 7: Figure S6, and the final results are presented in Table 1. Furthermore, the attributes of all genes located in the QTL regions are listed in detail in the Additional file 8: Table S2). Using these combined attributes, we identified 31 genes as the most likely candidates to regulate the traits controlled by *Qivr2-2*, *Qivr5*, *Qivr16*, *Qivr17-2* and *Qivr19* (listed in Table 2). These genes were further evaluated based on their known function from the literature and phenotypes in knock-out mouse mutants (see discussion).

**Table 1:** Candidate genes in mapped QTL intervals

***Qivr***	**Interval**	**Allele increasing survival**	**No of genes in interval**	**No of genes expressed during infection**	**No of genes with Indels (FS in coding region)**	**No of genes with SNPs (non-synonymous codons / stop codons)**	**Cis-eQTLs in non-infected lung**	**Differentially expressed btw B6/D2**
				**(up-/down- regulated)**				
*2-2*	56-68 Mb	D2	72	24 (19)	-	4 / 0	6	3
*5*	140-153 Mb	D2	179	83 (57)	-	9 / 0	13	10
*16*	64-78 Mb	B6	52	12 (7)	1^a^	5 / 0	4	4
*17-2*	30-44 Mb	B6	370	158 (104)	1^b^	58 / 4^c-f^	32	40
*19*	37– 45 Mb	D2	161	51 (30)	1^g^	14 / 1^h^	11	4

Up- or down-regulated genes were defined as genes that exhibited at least a 1.5 difference change in expression levels in lungs of infected mice compared to the non-infected controls. Genes were defined as differentially expressed exhibiting at least a 1.5 difference in expression changes in infected lungs of C57BL/6J and DBA/2J. Genes with sequence variations: ^a^*Robo1*, ^*b*^*Lst1*, ^c^*Brd2*, ^d^*H2-Ab1*, ^*e*^*H2-Bl*, ^f^*Rpp21*, ^g^*Tctn3*, ^h^*DhdpsI.* Abbreviations: FS: frame shift in coding region, SNP: single nucleotide polymorphism, cis-eQTL: cis regulated expression QTL, B6: C57BL/6J, D2: DBA/2J.

**Table 2:** GO-terms and functions observed in knock-out mice of 31 potential candidates from QTL intervals

***Qivr***	**Gene symbol**	**Gene description**	**Function**	**KO phenotype**	**Type polymorphism in ORF**
*Qivr2-2*	*Itgb6*	integrin beta 6	Integrin-mediated signaling pathway, inflammatory response, cell-matrix adhesion	Baldness associated with macrophage infiltration of skin, exaggerated pulmonary inflammation, impaired mucosal mast cell response to nematode infection.	ns (1)
*Qivr2-2*	*Ifih1*	interferon induced with helicase C domain 1	Response to virus, innate immune response, regulation of apoptosis, RIG-I-like receptor signaling pathway	Increased virus-associated morbidity and mortality, decreased cytokine response to several viral infection.	ns (5)
*Qivr5*	*Eif3b*	eukaryotic translation initiation factor 3, subunit B	Translation, translation initiation	NA	ns (1)
*Qivr5*	*Sdk1*	sidekick homolog 1 (chicken)	Cell adhesion	NA	ns (1)
*Qivr5*	*Eif2ak1*	eukaryotic translation initiation factor 2 alpha kinase 1	Negative regulation of translation, response to stress, negative regulation of cell proliferation, regulation of eIF2 alpha phosphorylation by heme	Enlarged heart size, abnormal red blood cell development, morphology, physiology with macrocytic anemia.	ns (1)
*Qivr5*	*Rnf6*	ring finger protein (C3H2C3 type) 6	Ubiquitin-dependent protein catabolic process, positive regulation of transcription, DNA-dependent	NA	ns (2)
*Qivr16*	*Robo1*	roundabout homolog 1 (Drosophila)	Cell differentiation, axon guidance, chemotaxis	Neonatal death, aphagia, delayed lung maturation and bronchial hyperplasia.	insertion
*Qivr16*	*Nrip1*	Nuclear receptor interaacting protein 1	Regulation of transcription	Female infertility due to ovulation failure. Male and female mice are smaller than wild-type littermates.	ns (1)
*Qivr16*	*Usp25*	ubiquitin specific peptidase 25	Ubiquitin-dependent protein catabolic process	NA	ns (1)
*Qivr17-1*	*Cryaa*	crystallin, alpha A	Negative regulation of apoptosis, negative regulation of caspase activity, lens fiber cell morphogenesis	Small lenses that develop progressive opacity beginning in the nucleus.	no
*Qivr17-2*	*Snf1lk/Sik1*	SNF1-like kinase	Negative regulation of transcription from RNA polymerase II promoter, regulation of cell differentiation, protein kinase cascade	NA	no
*Qivr17-2*	*March2*	membrane-associated ring finger (C3HC4) 2	Endocytosis, biological process	NA	no
*Qivr17-2*	*Tapbp*	TAP binding protein	Antigen processing and presentation of exogenous peptide antigen via MHC class I, TAP-dependent; defense response	Reduced and thermolabile MHC class I surface expression due to impaired peptide loading with stabilizing peptides, impaired T cell selection, altered NK repertoire, lower CD8+ T cell numbers, impaired responses to select class I-restricted antigens.	ns (1)
*Qivr17-2*	*H2-Oa*	histocompatibility 2, O region alpha locus	Antigen processing and presentation of peptide or polysaccharide antigen via MHC class II, regulation of T cell differentiation, Graft-versus-host disease, viral myocarditis	Abnormal antigen presentation via MHC class II, enhanced selection of CD4+ single positive thymocytes. Mice homozygous for a different knock-out allele show increased serum IgG1 levels.	ns (1)
*Qivr17-2*	*H2-DMa*	histocompatibility 2, class II, locus DMa	Antigen processing and presentation of exogenous peptide antigen via MHC class II, positive regulation of T cell differentiation, positive regulation of immune response, Graft-versus-host disease, viral myocarditis	Impaired antigen presenting cell function, poor IgG responses to T-dependent antigens, reduced numbers of mature CD4+ T cells, increased susceptibility to Leishmania major infection.	ns (2)
*Qivr17-2*	*Tap2*	transporter 2, ATP-binding cassette, sub-family B (MDR/TAP)	Antigen processing and presentation of exogenous protein antigen via MHC class Ib, TAP-dependent; positive regulation of T cell mediated cytotoxicity, protection from natural killer cell mediated cytotoxicity	No CD8+ T cells, although numbers of CD4+ T cells and B cells are normal.	ns (6)
*Qivr17-2*	*H2-Ob*	histocompatibility 2, O region beta locus	Antigen processing and presentation of peptide or polysaccharide antigen via MHC class II, Graft-versus-host disease, viral myocarditis	NA	ns (7)
*Qivr17-2*	*H2-Ab1*	histocompatibility 2, class II antigen A, beta 1	Antigen processing and presentation of peptide or polysaccharide antigen via MHC class II, Graft-versus-host disease, viral myocarditis	Depletion of mature CD4+ T cells, deficiency in cell-mediated immune responses, increased susceptibility to viral infections.	ns (9) stop_L
*Qivr17-2*	*H2-Aa*	histocompatibility 2, class II antigen A, alpha	Antigen processing and presentation of exogenous peptide antigen via MHC class II, positive regulation of T cell differentiation, Graft-versus-host disease, viral myocarditis	Lack of cell surface expression of MHC class II molecules on macrophages, decreased CD4-positive T cell number, increased CD8-positive T cell number, thymus hyperplasia, enlarged lymph nodes, altered splenocyte response to staphylococcal enterotoxin B.	ns (10)
*Qivr17-2*	*Lst1*	leukocyte specific transcript 1	Negative regulation of lymphocyte proliferation, immune response, cell morphogenesis	NA	ns (1) deletion
*Qivr17-2*	*Gtf2h4*	general transcription factor II H, polypeptide 4	Regulation of transcription, DNA-dependent	NA	ns (3)
*Qivr17-2*	*H2-T23*	histocompatibility 2, T region locus 23	Antigen processing and presentation of peptide antigen via MHC class I, Graft-versus-host disease, viral myocarditis	CD4^+^ T cells have enhanced responses after infection or immunization, are resistant to suppressor activity mediated by a subset of CD8^+^ T cells, but are more susceptible to NK cell lysis.	ns (3)
*Qivr17-2*	*H2-Bl*	histocompatibility 2, blastocyst	Antigen processing and presentation	NA	ns (4) stop_L
*Qivr17-2*	*Rpp21*	ribonuclease P 21 subunit (human)	tRNA processing	NA	ns (1) stop_G
*Qivr17-2*	*Trim26*	tripartite motif protein 26	Biological process	NA	ns (1)
*Qivr17-2*	*Pla2g7*	phospholipase A2, group VII (platelet-activating factor acetylhydrolase, plasma)	Inflammatory response, lipid catabolic process	NA	ns (1)
*Qivr17-2*	*Cyp39a1*	cytochrome P450, family 39, subfamily a, polypeptide 1	Lipid metabolic process, oxidation reduction	NA	ns (2)
*Qivr-19*	*Sorbs1*	sorbin and SH3 domain containing 1	Transport, focal adhesion assembly, positive regulation of establishment of protein localization in plasma membrane	Decreased triglyceride levels, altered glucose homeostasis, decreased white blood cells and resistance to developing glucose intolerance induced by a high fat diet.	ns (6)
*Qivr-19*	*Tctn3*	tectonic family member 3	Apoptosis	NA	ns (1) insertion (2)
*Qivr-19*	*Hps1*	Hermansky-Pudlak syndrome 1 homolog (human)	Positive regulation of natural killer cell activation, secretion of lysosomal enzymes	Hypopigmentation and increased bleeding time. Impaired natural killer cell function, reduced secretion of kidney lysosomal enzymes, abnormal retinofugal neuronal projections characterize some alleles.	ns (6)
*Qivr-19*	*Dnmbp*	dynamin binding protein	Intracellular signaling cascade, regulation of Rho protein signal transduction	NA	ns (3)

The genotype of C57BL/6J was used as reference for the sequence polymorphisms. The knockout mutant phenotype was identified in the MGI Mouse Genome Database. Gene *Fign* (fidgetin) fulfilled most criteria but was found to be expressed only at late times p.i. (after day 14) and was therefore omitted from the candidate gene list. Abbreviations: KO: knockout mutant, NA: not analyzed, SNP: single nucleotide polymorphism, ns: non-synonymous codons, ORF: open reading frame, stop_G: stop codon gained, stop_L: stop codon lost.

## Discussion

DBA/2J and C57BL/6J mice have been shown previously to differ largely in their susceptibility to H1N1 (PR8M) influenza A virus [9,18]. Here, we expanded these studies and utilized the BXD recombinant inbred set of mouse strains to map the genomic regions that are responsible for differences in these two mouse strains. We monitored three phenotypic traits, body weight over time, survival over time and mean time to death to identify quantitative trait for influenza resistance. Two significant QTLs, *Qivr5* and *Qivr19*, were found on chromosomes 5 and 19, respectively. Furthermore several suggestive QTLs, *Qivr2*-2, *Qivr16* and *Qivr17-2* were observed in at least two traits and at two days on chromosomes 2, 16 and 17, respectively. Composite mapping revealed an additional almost significant QTL at distal chromosome 16, *Qivr16*.

A similar analysis for host resistance to influenza has been performed previously after infecting 66 BXD strains with H5N1 influenza virus. This study reported three significant QTLs on chromosomes 2, 7, and 17 [12]. Thus, none of these significant QTLs overlaps with the QTLs identified in our analysis. Five of the strains that were resistant (all infected mice survived) in our study were also resistant in the study of [12] where a total of 14 strains were found to be resistant. Five strains that were highly susceptible in our study (100% of infected mice died) were also highly susceptible in the study by [12] of a total of 26 susceptible strains. Furthermore, five strains that were resistant in our study were susceptible in the study by [12]. Thus, there is also not much overlap between the two studies with respect of susceptible and resistant strains. The differences between the two studies are most likely explained by the use of two different influenza virus subtypes. The H1N1 virus from our study represents a subtype with a monobasic hemagglutinin (HA) cleavage site, whereas the H5N1 which was used in the study by Boon et al. is a subtype with a polybasic HA cleavage site. The cellular tropism of these two subtypes for virus replication and processing is quite different, because monobasic viruses are dependent on cell-specific proteases for the processing of the HA whereas polybasic subtypes can be processed by more ubiquitously expressed host proteases, *e.g.*[27-32]. Therefore, the contribution of host factors to susceptibility may be different between H1 and H5 containing virus subtypes.

Another study described the genetic mapping of susceptibility and resistance factors after infecting a panel of 29 AxB / BxA congenic strains with a mouse-adapted H3N2 influenza virus [33]. The AxB / BxA congenic strains were generated from a cross of susceptible A/J and resistant C57BL/6J parental mouse strains. The authors found three major QTLs on chromosomes 2, 6 and 17. The QTL on chromosome 17 overlaps with the *Qivr17-2* locus which we found in our study. Furthermore, the candidate gene *Pla2g7* that was identified in their study was also detected as candidate gene in our analysis (see below).

The influence of genetic factors determining the host response to H1N1 influenza virus infections was also examined in mice of the pre-Collaborative Cross collection [34]. In this study, gene expression levels in extreme responders were used to identify expression QTLs (eQTL). One gene that exhibited a cis-eQTL, *Sik1* (salt inducible kinase 1), was located in the *Qivr17-2* interval from our study, and we also identified it as potential quantitative trait gene (Table 2). This gene is associated with the GO terms ‘negative regulation of transcription from RNA polymerase II promoter, regulation of cell differentiation, and protein kinase cascade’. However, no specific infection-related functions have been yet described for this gene.

One of the most interesting findings in our study was the time-dependent effect of QTLs which we observed in the body weight and survival traits. The peak QTLs for the two significant QTLs, *Qivr5* and *Qivr19*, were found at different times p.i., day 6 and day 8, respectively. In addition, the effects of both QTLs were not only evident at the times p.i. where they exerted the significant peak QTL signals but also several days before and after the peak. Furthermore, for the suggestive QTLs, also time-dependent effects were observed. These results suggest that the causal genes underlying different QTLs act at different time points of the host defense.

Most interestingly, *Qivr5* as well as *Qivr19* represent a positive influence on body weight, survival and MTTD from the DBA/2J haplotype, the susceptible strain. These findings indicate that genomic regions from the susceptible parent are able to increase resistance when combined with the resistant parental genome. We are now analyzing several BXD strains that were more resistant than the parental C57BL/6J mice in more detail. One possible mechanism to explain such an effect may be that an activator (secreted ligand or transcription factor) is expressed in susceptible DBA/2J mice but the corresponding target (receptor or regulated gene) is mutated. On the other hand, in C57BL/6J mice, the target but not the activator may be mutated. If the wild type alleles are now coming together in a BXD strain, the functional activator finds its functional target and thereby an increased resistance state is achieved.

Both composite and interaction mapping revealed many genetic interactions between C57BL/6J and DBA/2 J alleles. Thus, many genomic regions from the parental strains are able to contribute to the host response and this effect depends strongly on the allele combinations in the respective QTLs. These observations may be studied further in double congenic mouse lines.

We subsequently analyzed the five QTL intervals, *Qivr2*-2, *Qivr5*, *Qivr16*, *Qivr17-2* and *Qivr19* in more detail to identify genes that may be causal for resistance or susceptibility. In total, 830 genes are located in these intervals. We narrowed down the total number of genes to 31 candidates (Table 2) by using additional information, such as temporal expression after PRM8 infection (Pommerenke et al., PLoS ONE, in press), cis-eQTLs in non-infected lungs [26], differences in expression between DBA/2J and C57BL/6J [23], and sequence variants in the coding regions.

*Qivr5* contains the candidate gene *Eif2ak1* (eukaryotic translation initiation factor 2 alpha kinase 1) that is a member of eIF2alpha kinases which have been associated with anti-viral host responses [35]. Boon et al. described another eIF2alpha kinase, *Eif2ak2* / *Pkr* (eukaryotic translation initiation factor 2-alpha kinase 2), in the *Qirv17* locus after infection with influenza H5N1 [12]. *Eif2ak2* plays a critical role in modulating immunoglobulin expression during RSV infection. In addition *Eif2ak2* knock-out mouse mutants are more susceptible to influenza infections [36,37]. We have initiated the generation of a congenic mouse lines for the chromosome 5 interval to verify and further characterize the effect of this region for resistance to influenza infection.

*Qivr2-2* contains two candidate genes, *Itgb6* (integrin beta 6) and *Ifih1* (interferon induced with helicase C domain 1), with known functions in the host defense to viral infections. *Itgb6* mouse knock-out mutants exhibit severe pneumonia and an increase in granulocyte recruitment to the lung [38]. The protease-activated receptor 1-mediated enhancement of *Itgb6-*dependent TGF-beta activation has been proposed to represent one mechanism by which activation of the coagulation cascade contributes to the development of acute lung injury [39]. The *Ifih1* gene is also known as MDA5 (Melanoma Differentiation-Associated protein 5). IFIH1 is part of the RIG-I-like receptor (RLR) family, which function as pattern recognition receptors and are activated upon binding of virus dsRNA [40]. IFIH1 functions as cytosolic receptor that leads to the selective activation of type I IFN genes and is indispensable for sustained expression of IFN in response to paramyxovirus infection [41,42]. *Ifih1* mutant knock-out mice exhibit an impaired response to different viral pathogens [43,44].

*Qivr16* contains two potential genes with known functions in the host defense and lung function, *Robo1* (roundabout homolog 1 (Drosophila)) and *Nrip1* (nuclear receptor interacting protein 1). DBA/2J mice carry a frame shift mutation in the *Robo1* gene which might lead to an impaired function of the encoded protein. *Robo1* has been described to be involved in guidance and migration of axons, myoblasts, and leukocytes in vertebrates (*e.g.*[45-47]*)* but is also expressed in the developing lung [48]. *Robo1* knock-out mutants exhibit a delayed lung maturation and bronchial hyperplasia. The latter results suggest that *Robo1* may be involved in maintaining proper lung function and it may become essential when lung epithelium is destroyed during an influenza infection. *Nrip1/Rip140* functions as a co-activator for cytokine gene promoter activity via direct protein-protein interactions with the NFkappaB subunit RelA and histone acetylase cAMP-responsive element binding protein (CREB)-binding protein (CBP) [49]. It is involved in modulating pro-inflammatory responses in macrophages [50].

*Qivr17-2* represents a positive influence of the C57BL/6 J genotype on body weight, survival and MTTD. This QTL is located in a gene-rich region which carries many genes that are involved in the host immune response, in particular the *H2* histocompatibility genes which are involved in antigen presentation [51]. Therefore, many candidate genes are found in the *Qivr17-2* region. The *Lst1* (leukocyte specific transcript 1) gene is of special interest because the DBA/2J allele mice carries a single nucleotide deletion in the first exon resulting in a frame shift of the open reading frame. This mutation most likely results in a non-functional Lst1 protein in DBA/2 J mice. We confirmed the presence of the deletion by sequencing the parental DBA/2J and some BXD strains carrying the DBA/2J allele. The wild type allele was confirmed in C57BL/6J mice and in some BXD strains carrying the C57BL/6J allele. In humans, *LST1* plays a role in the regulation of the immune response to inflammatory diseases such as rheumatoid arthritis, microbial infection or Rubella vaccine-induced immunity [52-55]. Also, *Lst1* is up-regulated after influenza A infection in C57BL/6J mice starting at day 2 and exhibits a strong peak of expression at day 8 p.i. Pommerenke et al., 2012 (Pommerenke, C., E. Wilk, B. Srivastava, A. Schulze, N. Novoselova, R. Geffers, and K. Schughart. 2012. Global transcriptome analysis in influenza-infected mouse lungs reveals the kinetics of innate and adaptive host immune responses. PLoS ONE. 7:e41169.). Thus, the expression profile and known functions of *Lst1* fit well with a possible critical role for the host defense to influenza A virus. We initiated the generation of knock-out mice to evaluate the role of *Lst1* in more detail. In addition, a second, most interesting candidate, *Pla2g7* (phospholipase A2, group VII (platelet-activating factor acetylhydrolase, plasma)) was identified in the *Qivr17-2* interval. In humans, increased activities of certain variants of PLA2G7 were associated with early coronary atherosclerosis and with endothelial dysfunction, but the gene may also exert an anti-inflammatory function [56-60]. The *Pla2g7* gene was also identified as a potential candidate gene for susceptibility against infections with H3N2 influenza virus [33]. *Pla2g7* expression levels in susceptible A/J mice were higher than in resistant C57BL/6J mice after infection with H3N2 virus [33]. We also showed previously that *Pla2g7* exhibits a cis-eQTL between C57BL/6J and DBA/2J in non-infected lungs where the DBA/2J allele shows high levels of expression [26]. *Tnfrsf21* which was identified by [33] as potential candidate of *Qivr17-2* also exhibits a cis-eQTL in non-infected BXD mice [26] but was not found to be regulated in C57BL/6 mice after infection (data not published). *Tapbp* (TAP binding protein) plays a major role in the antigen processing and MHC class I presentation by stabilizing the TAP peptide transporter, *e.g.*[61-65]. Also, *Tap2* (transporter 2, ATP-binding cassette, sub-family B (MDR/TAP)) gene is involved in antigen processing and presentation [63,66]. *Gtf2h4* (general transcription factor II H, polypeptide 4) encodes a general transcription factor. Recruitment and activation of *Gtf2h4* represents a rate-limiting step for the emergence of HIV from latency and sequence variants have been associated with multiple sclerosis [67-69].

Within the *Qivr19* interval, only one gene, *Hps1* (Hermansky-Pudlak syndrome 1 homolog (human)), has been associated with the host responses to infection. Mice carrying a natural mutation in the *Hps1* gene showed an increased inflammatory response in alveolar macrophages after intranasal challenge with LPS [70].

The GeneNetwork database allows searching for other phenotypic traits that exhibit a genome-wide significant (LRS ≥ 18) within the *Qivr* intervals identified by our study. Two phenotypic traits, related to neuronal responses (trait ID 11285) and body weight changes (trait ID 12838), are located to the *Qivr16* locus. Also, the Qivr17-2 interval contained significant QTLs for other traits. Two traits are related to host infectious diseases, ‘Ectromelia virus survival’ (ID 12672) and ‘Chlamydia psittaci (6BC) infection response’ (ID 11025) and four traits are associated with immune cell responses (ID 10201, 10466, 10238, 10236). In addition two traits described seizure responses (ID 10388, 10507), and one trait has not been disclosed yet (ID 13920). Within the early time chromosome 10 interval, three other traits exhibit their most significant QTLs: ‘3a,5a-THDOC in blood plasma 3 days after cycle 5 of chronic intermittent air vapor’ (ID13027) and two non-disclosed traits. The first trait may relate to stress responses in the central nervous system (ID 13292 and 13846).

## Conclusions

The mapping of resistance and susceptibility loci in the BXD population revealed several new QTLs and potential gene candidates that may be critical for the host defense against influenza A virus infection. Body weight and survival QTLs showed a time-dependent profile indicating that the genetic factors in these QTLs are important for the host response in a temporal dynamic fashion. Five QTL regions were examined in detail, and we identified several possible candidate genes that may be critical for the host response to influenza A infections in humans.

## Methods

### Animals

The mouse inbred strains C57BL/6J, DBA/2J and B6D2F1 were delivered from Janvier, France. Recombinant inbred mouse strains BXD were purchased from three different sources: The Jackson Laboratory, the University of Tennessee Health Science Center (Memphis, TN) and from Harlan, The Netherlands. For the analysis, mice were transferred to the animal facility in Braunschweig and adapted for at least two weeks to the new environment before starting experiments. Animals were maintained under specific pathogen free conditions. All experiments in mice were approved by an external committee and according to the national guidelines of the animal welfare law in Germany (‘Tierschutzgesetz in der Fassung der Bekanntmachung vom 18. Mai 2006 (BGBl. I S. 1206, 1313), das zuletzt durch Artikel 20 des Gesetzes vom 9. Dezember 2010 (BGBl. I S. 1934) geändert worden ist.’). The protocol used in these experiments has been reviewed by an ethics committee and approved by the ‘Niedersächsisches Landesamt für Verbraucherschutz und Lebensmittelsicherheit, Oldenburg, Germany’, according to the German animal welfare law (Permit Numbers: 33.42502/04-108/06, 33.9.42502-04-051/09).

### Virus and infection of mice

The mouse-adapted influenza strain A/Puerto Rico/8/34 (H1N1; PR8M, [18] and references therein) was used for all infection studies. Stocks were prepared by infection of 10-day-old embryonated chicken eggs. After mice were anesthetized by intra-peritoneal injection of Ketamin-Xylazine solution in sterile NaCl (50 mg/ml Ketamine, Invesa Arzneimittel GmbH, Freiburg; 2% Xylazine, Bayer Health-Care, Leverkusen) with a dose adjusted to the individual body weight, mice were infected intranasally with 2 × 10^3^ FFU of PR8M in 20 μl of sterile phosphate-buffered saline. Mice were assayed daily for body weight (determined as % of initial weight at day 0) and mortality during 13 days p.i. We used death as the end point for survival. Mice were sacrificed if body weight loss exceeded 25%. It should be noted that for mice that did not show any signs of body weight loss over the entire time period after infection, we do not have additional parameters to verify that they have indeed been infected. However, these cases were very few. In addition, we have ample experience with this infection method and the failure rate, for example with the DBA/2J strain, is less that 5%.

### Data handling and statistical analysis

In total, 283 BXD mice and 127 mice from the parental strains or F1 generation were used for the infection experiments. In total 53 BXD strains were infected with an average of 5 mice per strain. We performed all primary calculations using simple features and functions of Microsoft Excel. Three sets of analysis were performed for the following variables: (1) body weight in percentage from starting weight (day 0) using the strain medians to exclude outliers; (2) survival by calculating the survival rate of each strain from day 7 to 13, (3) mean time to death in days. For statistical analyses, tests and visualization we used R, a free software environment for statistical computing and graphics (http://www.R-project.org). In order to test for batch effects or other co-factors, we visualized the data using multidimensional scaling based on the Sammon mapping method [71]. No clusters with respect to any of the co-factors age, weight at day 0, experimenter, time of infection, or source of mice could be found in the visualizations.

### QTL mapping

QTL mapping was performed using the web-based complex trait analysis available on the GeneNetwork website (http://www.genenetwork.org) and the mapping module to analyze phenotypes in context of mouse genotypic differences. Interval mapping evaluates potential QTLs at regular intervals and estimates the significance at each location with a graphical representation of the likelihood ratio statistics (LRS) using 2000 permutation tests [19,22]. LRS values may be converted to LOD scores by dividing by 4.61. For the two locus model the following equation was used: *Var = Q1 + Q2 + Q1xQ2 + e*, where *Var* = the between-strain mean variance in the trait, *Q1* and *Q2* are makers tightly linked to the loci, *Q1XQ2* is the ‘additive-by-additive’ epistatic interaction term, and *e* is the residual error. The original data sets can be obtained at http://www.genenetwork.org with the following identification numbers: body weight: 13005 to 13017; survival: 13000 to 13004 and mean time to death: 12996. We performed full genome scans for epistatic interactions using the pair-scan module that is implemented in GeneNetwork. This module exploits the direct global optimization algorithm developed by [72]. The code compares the fit (as measured by LRS scores) for a purely additive model, a purely epistatic model, and the full model. The code also implements a permutation test (n = 500) and this enabled us to estimate the empirical p value of the alternative models.

### Candidate gene discovery

The QTL region analysis was initially performed using the QTLminer which has been implemented in GeneNetwork [25]. By using the automatic function of GeneNetwork we identified significant cis-QTLs with LRS higher than 18 at a genome-wide p-value of < 0.05. Additionally the genes mapped within the analyzed QTLs were surveyed by the National Center for Biotechnology Information (NCBI) Entrez Gene website (http://www.ncbi.nlm.nih.gov/sites/entrez?db=gene) and the Jackson Laboratory's MGI Mouse Genome Database project (http://www.informatics.jax.org/) to identify potential candidate genes. The GeneRIF database (http://www.ncbi.nlm.nih.gov/projects/GeneRIF/GeneRIFhelp.html) was used as a primary source to search for known gene functions and corresponding citations.

All sequence variants between B6 and D2 parental genomes (SNPs, indels) were extracted by using a comparative analysis that relies on approximately 100x whole genome shot gun of DBA/2J [73]. All of these sequence data are available at http://ucscbrowser.genenetwork.org/, the GeneNetwork Variant Browser (http://www.genenetwork.org/webqtl/main.py?FormID=snpBrowser), and the NCBI Short Read Archive (18 files total, e.g., SRX037575, SRX013980, SRX013299, SRX012582, SRX012581, SRX012580); http://www.biomedcentral.com/1471-2105/11/S4/O7.

## Competing interests

The authors declare that they have no competing interests.

## Authors’ contributions

TN, MH and KS conceived and designed the experiments. TN and MH infected the mice and collected the data. HK, RA, RW and KS performed and interpreted the QTL analysis. FK performed the analysis of environmental and experimental factors. SS and LL provided BXD mice for experimental studies. HK, RW and KS wrote the manuscript. All authors read and approved the final manuscript.

## Authors’ information

Tatiana Nedelko and Heike Kollmus contributed equally as first authors.

Robert W. Williams and Klaus Schughart contributed equally as senior authors.

## Supplementary Material

Additional file 1 **Table S1.** Body weight loss from day 1 until day 7 for all strains showing the mean values, SEM per day and number of mice per strain analyzed. Click here for file

Additional file 2 **Figure S1.** Differential susceptibility to influenza A infection among different BXD strains, parental strains and F1 generation. Rank-ordered strain distribution pattern illustrating the percentage of surviving mice per strain after influenza A infection for 53 BXD, parental DBA/2J and C57BL/6J strains and F1 (B6D2F1) mice for day 7 (A, trait ID: 13001) and day 8 (B, trait ID: 13002). Rank-ordered strain distribution pattern showing mean time to death for 53 BXD, parental DBA/2J and C57BL/6J strains and F1 (B6D2F1) mice (C, trait ID: 12996). Click here for file

Additional file 3 **Figure S2.** Principal component analysis of all body weight traits. Percent contribution of principal components to the total variance for body weight traits from day 1 until day 7 p.i. PC1 contributes about 80% and PC2 about 10% to the total variance. Click here for file

Additional file 4 **Figure S3.** Body weight changes in mock-infected C57BL/6J and DBA/2J mice. Female DBA/2J (n=4) and C57BL/6J (n=3) mice were intranasally inoculated with 25 μl PBS under anesthesia. Body weight changes for each group of treated mice at various days p.i. is shown with reference to the starting weight (% body weight). Data represent mean values +/- SEM. Click here for file

Additional file 5 **Figure S4.** Genome-wide linkage analysis for survival after H1N1 infection at days 9 and 10 p.i. Analysis of survival from day 9 (A) and day 10 (B) after PR8 infection revealed suggestive QTLs on chromosomes 5, 16, 17 and 19. Interval mapping is shown across the entire genome as blue line representing the LRS scores; green line: DBA/2J alleles increase trait values; red line: C57BL/6J alleles increase trait values. Upper x-axis shows mouse chromosomes, lower x-axis shows physical map in mega bases for each chromosome, y-axis represents LRS score. The horizontal lines mark the genome-wide significant thresholds at p0.05 (red line) and suggestive thresholds at p0.37 (gray line). Click here for file

Additional file 6 **Figure S5.** Pair Scan analysis of trait 13002. The table provides the breakdown of trait 13002 as an example for a pair scan analysis study that compares the fit of the alternative models. An analysis of this type can be regenerated rapidly on any of the traits presented in our manuscript. Click here for file

Additional file 7 **Figure S6.** Scheme to identify potential candidate genes in the analyzed QTL intervals. The detailed steps are described in the main manuscript. Click here for file

Additional file 8 **Table S2.** List of genes in the QTL intervals that were studied in detail. The QTLminer tool in GeneNetwork was to identify all genes in the QTL intervals and to obtain associated GO terms and KEGG pathways. All annotated genes within the QTL intervals that were expressed between day 1 and day 60 after infection of C57BL/6J mice with PR8M virus were further analyzed for the following attributes: genes that were up- or down-regulated in infected lungs after PR8M infection, genes carrying an insertion or deletion or a non-synonymous nucleotide change in the open reading frame, genes exhibiting a cis-expression QTL in the lung, and genes that were differentially expressed in C57BL/6J and DBA/2J mice in infected lungs between day 1 and day 8 p.i. Abbreviations: FS: frame shift in coding region, SNP: single nucleotide polymorphism, cis-eQTL: cis-regulated expression QTL, B6: C57BL/6J, D2: DBA/2J, ns: non-synonymous codons, stop_G: stop codon gained, stop_L: stop codon lost, DEL: deletion, INS: insertion; ns (…): number of non-synonymous codons. Legends: Expression d0-d60, log2 ≥8 at any day during infection; No: log2 8; ?: no expression data available. Regulation during infection (d0-d60): + - up or down regulation, genes that exhibited at least a 2-fold difference in changes of the expression levels in lungs of infected mice compared to non-infected controls; (+)(-): up-or down-regulation, genes that exhibited at least a 1.5-fold difference in changes of the expression levels in lungs of infected mice compared to the non-infected controls; no: no regulation. SNP: ns (.) number of non-synonymous codons; STOP_L: Stop codon lost, STOP_G: Stop codon gained; no: no SNPs between B6 and D2. Indel: DEL: Deletion; INS: Insertion; no: no INDEL. cisQTL: yes (…): LRS value has to be greater than ≥18; no: no QTL or LRS 18. diff expressed (B6-D2) during infection: Genes were defined as differentially expressed (log2 ≥8) at any day in infected lungs of C57BL/6J and DBA/2J; + -: Genes were defined as differentially expressed exhibiting at least a 2.0 difference in changes of the expression levels in infected lungs of C57BL/6J and DBA/2J; (+)(-) differentially expressed: Genes were defined as differentially expressed exhibiting at least a 1.5 difference in changes of the expression levels in infected lungs of C57BL/6J and DBA/2J; no: no regulation or not expressed; Score: the different attributes were counted. Click here for file
