# Supplementary figures and images for: Distinct gene loci control the host response to influenza H1N1 virus infection in a time-dependent manner
Source: BMC Genomics. 2012 Aug 20;13:411. doi: 10.1186/1471-2164-13-411 (PMC3479429; doi:10.1186/1471-2164-13-411)

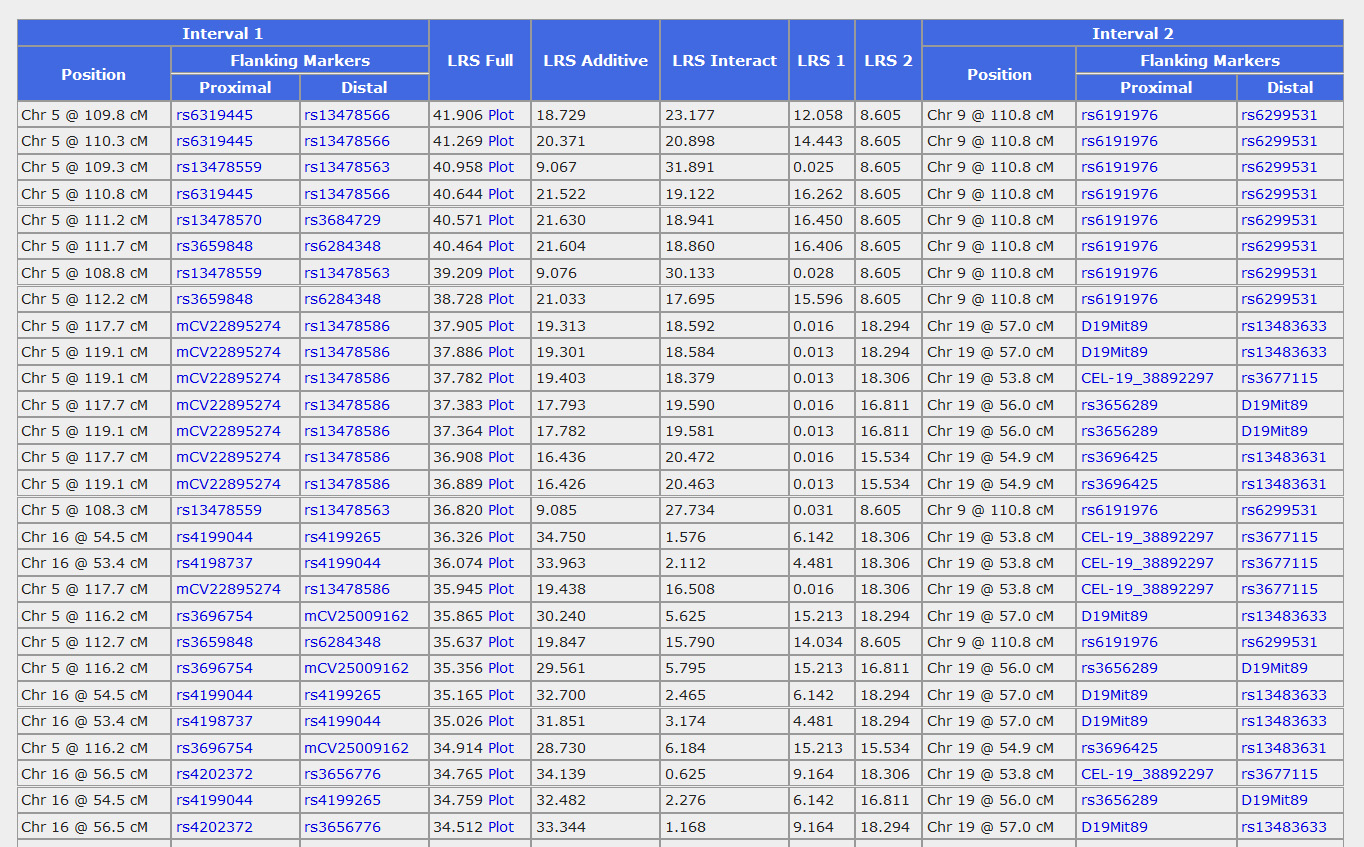

Supplement: Additional file 1 — Table S1. Body weight loss from day 1 until day 7 for all strains showing the mean values, SEM per day and number of mice per strain analyzed. [file 1471-2164-13-411-S1.jpeg]

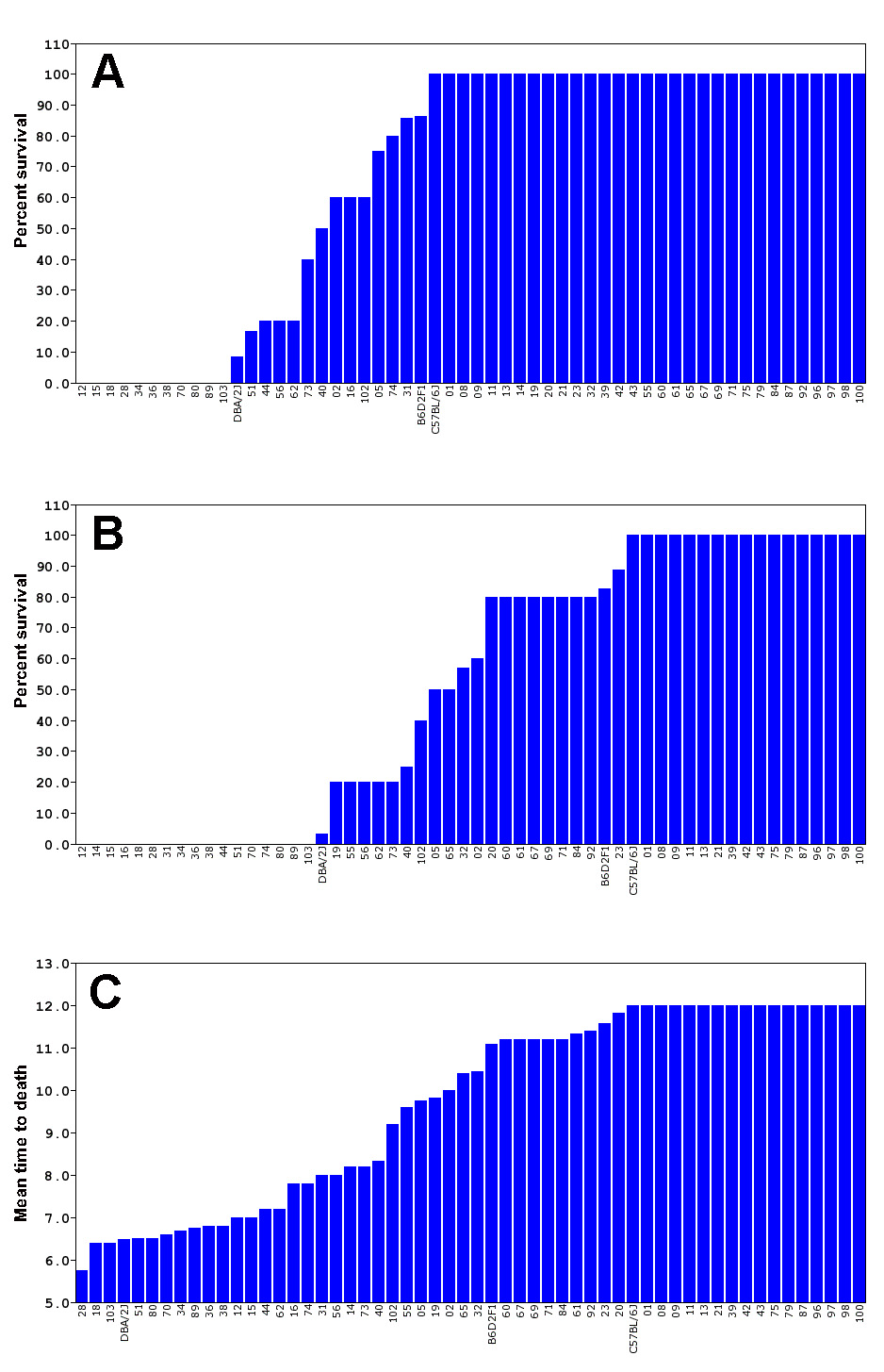

Supplement: Additional file 2 — Figure S1. Differential susceptibility to influenza A infection among different BXD strains, parental strains and F1 generation. Rank-ordered strain distribution pattern illustrating the percentage of surviving mice per strain after influenza A infection for 53 BXD, parental DBA/2J and C57BL/6J strains and F1 (B6D2F1) mice for day 7 (A, trait ID: 13001) and day 8 (B, trait ID: 13002). Rank-ordered strain distribution pattern showing mean time to death for 53 BXD, parental DBA/2J and C57BL/6J strains and F1 (B6D2F1) mice (C, trait ID: 12996). [file 1471-2164-13-411-S2.jpeg]

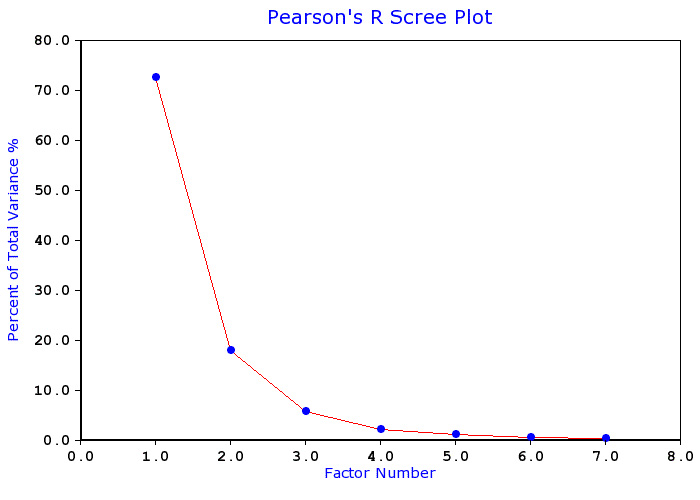

Supplement: Additional file 3 — Figure S2. Principal component analysis of all body weight traits. Percent contribution of principal components to the total variance for body weight traits from day 1 until day 7 p.i. PC1 contributes about 80% and PC2 about 10% to the total variance. [file 1471-2164-13-411-S3.jpeg]

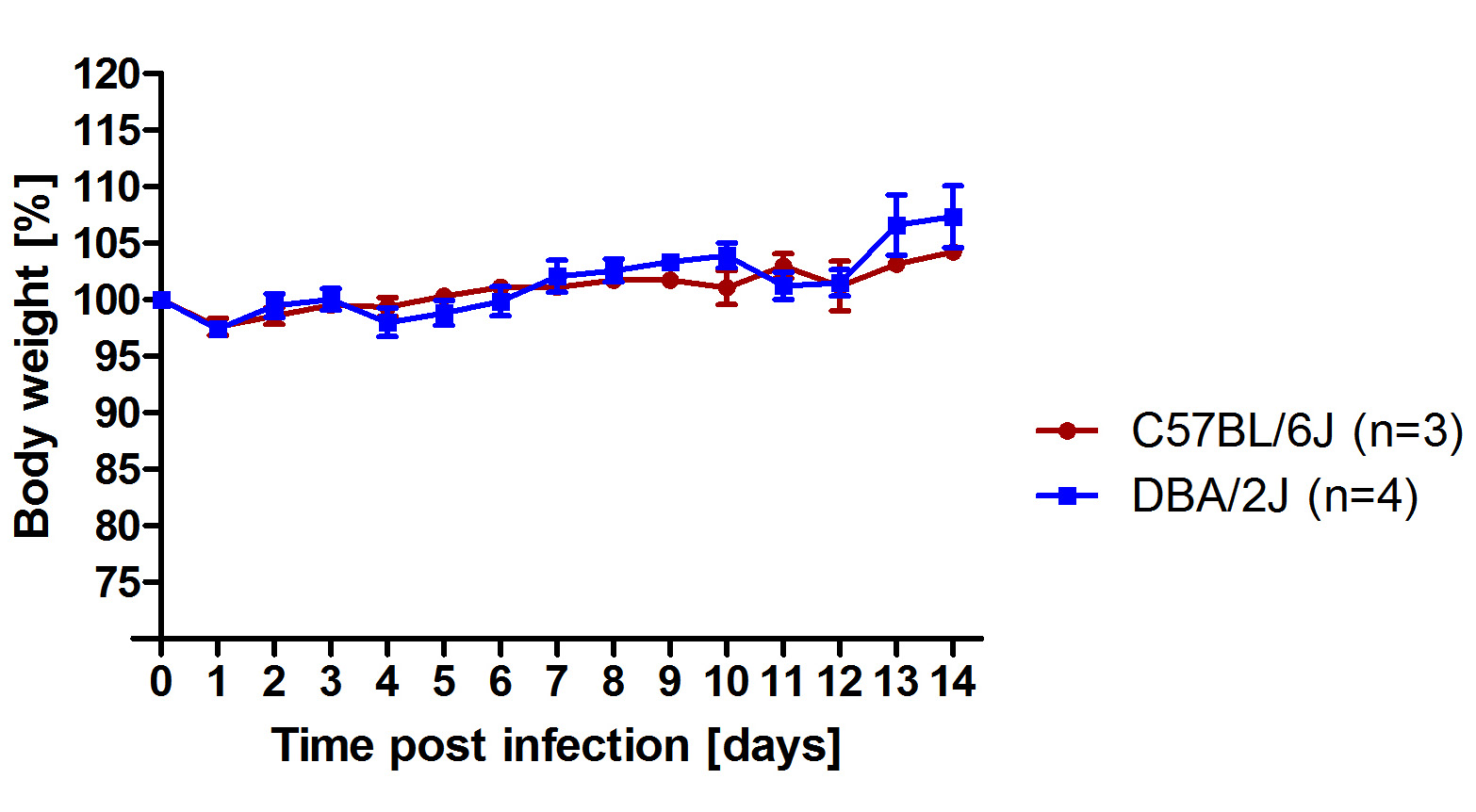

Supplement: Additional file 4 — Figure S3. Body weight changes in mock-infected C57BL/6J and DBA/2J mice. Female DBA/2J (n=4) and C57BL/6J (n=3) mice were intranasally inoculated with 25 μl PBS under anesthesia. Body weight changes for each group of treated mice at various days p.i. is shown with reference to the starting weight (% body weight). Data represent mean values +/- SEM. [file 1471-2164-13-411-S4.jpeg]

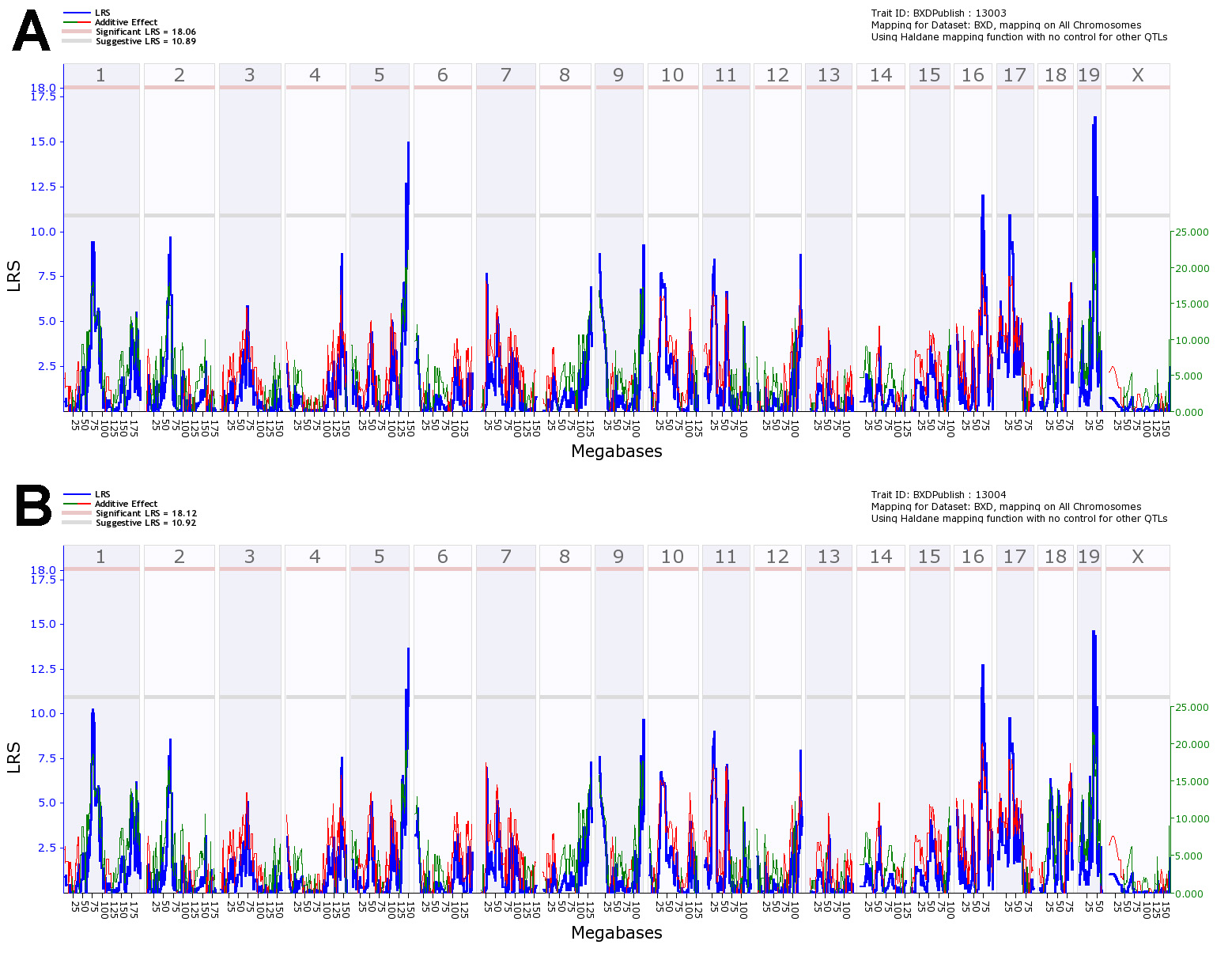

Supplement: Additional file 5 — Figure S4. Genome-wide linkage analysis for survival after H1N1 infection at days 9 and 10 p.i. Analysis of survival from day 9 (A) and day 10 (B) after PR8 infection revealed suggestive QTLs on chromosomes 5, 16, 17 and 19. Interval mapping is shown across the entire genome as blue line representing the LRS scores; green line: DBA/2J alleles increase trait values; red line: C57BL/6J alleles increase trait values. Upper x-axis shows mouse chromosomes, lower x-axis shows physical map in mega bases for each chromosome, y-axis represents LRS score. The horizontal lines mark the genome-wide significant thresholds at p0.05 (red line) and suggestive thresholds at p0.37 (gray line). [file 1471-2164-13-411-S5.jpeg]

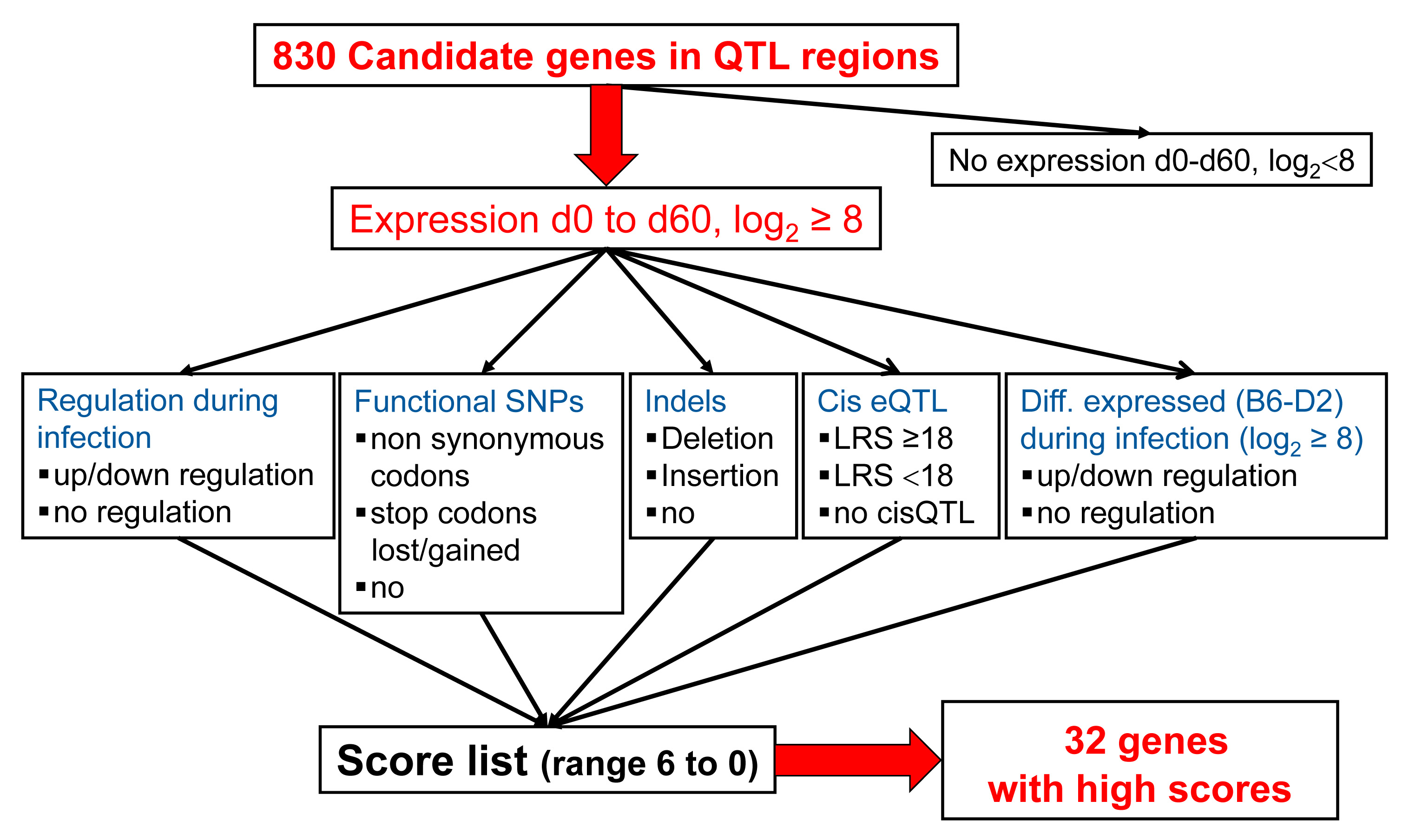

Supplement: Additional file 6 — Figure S5. Pair Scan analysis of trait 13002. The table provides the breakdown of trait 13002 as an example for a pair scan analysis study that compares the fit of the alternative models. An analysis of this type can be regenerated rapidly on any of the traits presented in our manuscript. [file 1471-2164-13-411-S6.jpeg]
